# Diagnosis and comprehensive therapy for cutaneous neuroendocrine carcinoma of the external auditory canal: a case report and literature review^☆^

**DOI:** 10.1016/j.bjorl.2016.02.005

**Published:** 2016-04-21

**Authors:** Qian Xiu, Xue-Ju Wang, Dong-Dong Zhu, Cui-Da Meng

**Affiliations:** a China-Japan Union Hospital of Jilin University, Department of Otolaryngology, Head and Neck Surgery, Changchun, China; b China-Japan Union Hospital of Jilin University, Department of Pathology, Changchun, China

## Introduction

Cutaneous neuroendocrine carcinoma (cNEC) is a rare, aggressive, malignant cutaneous tumor that was first reported by Toker^1^ in 1972. This tumor was described as an uncommon skin lesion that showed a trabecular pattern of tumor cell growth. The cNEC is also referred to as Merkel cell carcinoma because it is generally thought to originate from Merkel cells in the basal layer of the epidermis. The most common locations for cNEC are in the head and neck in areas such as the nasal cavity, lymph node, and salivary glands.^2^ According to large reviews, local recurrence develops in 25–30%, regional disease in 52–59%, and distant metastatic disease in 34–36% of cNEC cases.^3^ Local wide excision is the recommended primary treatment for regional disease, and postoperative radiation and elective lymph node dissection may minimize locoregional recurrence. Herein, we present a single case of cNEC located at the external auditory canal; only 4 cases of this rare tumor site have been reported worldwide according to the best of our knowledge. This case was successfully controlled with local excision and postoperative radiation therapy.

## Case report

**Figure 1:**
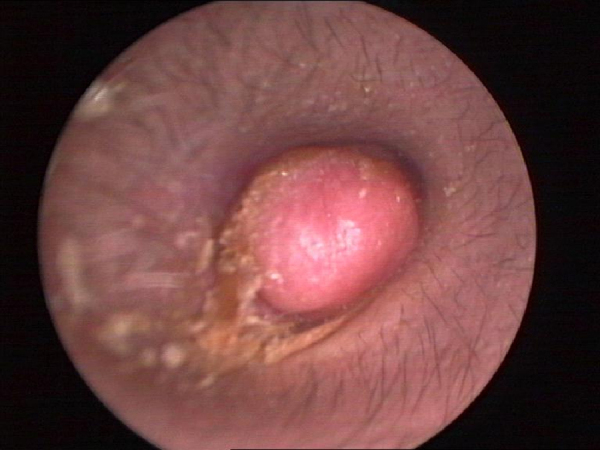
Endoscope test showing a pink, ovary-shaped neoplasm with a smooth surface. The tympanic membrane was covered without adhering to the adjacent structure.

**Figure 2:**
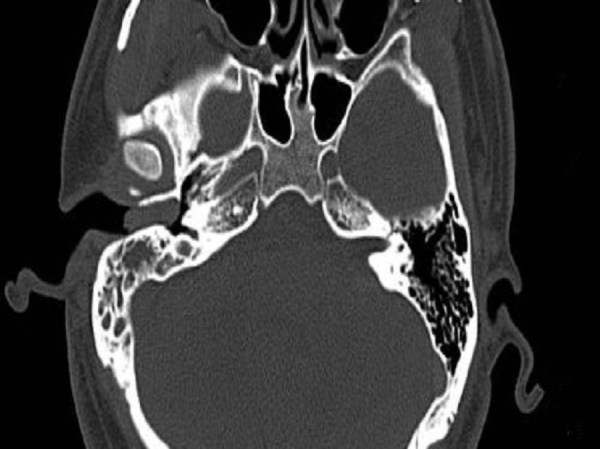
Axial CT scan before surgery showing a well-circumscribed strip of solid mass in the right external auditory canal without bony erosion and a medium-density area in the tympanic antrum.

**Figure 3:**
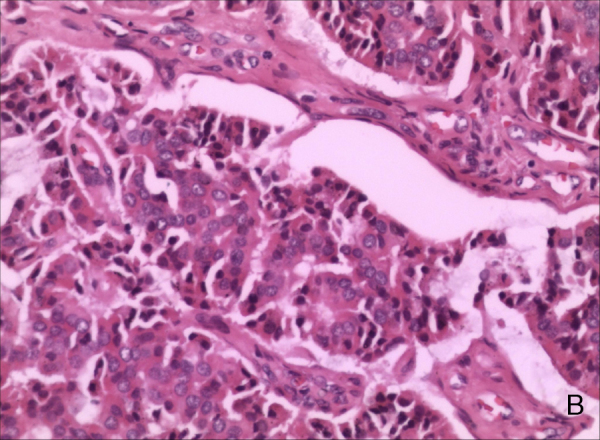
Vesicular nuclei, eosinophilic cytoplasm and high-density fragments could be seen in some cells (hematoxylin–eosin stain, magnification 200×).

**Figure 4:**
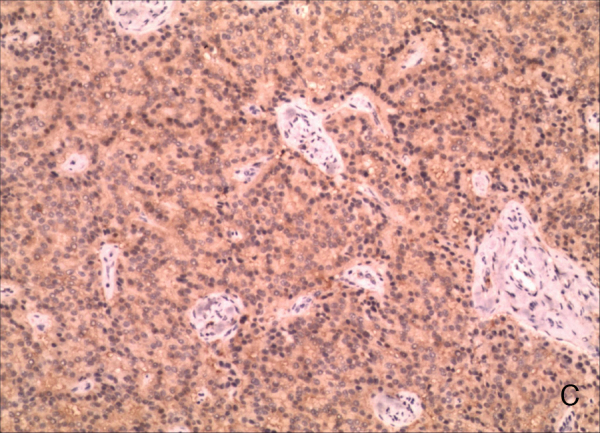
Immunohistochemistry for neuron-specific enolase (NSE) (magnification 100×).

A 26-year-old male presented with a one-year history of right external auditory canal itching, hearing loss, intermittent pain and 1 month of watery secretion. A clinical examination revealed a 1.04 cm in diameter, pink, ovary-shaped nodule with a smooth surface that was noted in his right external auditory canal covering the tympanic membrane (Fig. 1). Pure-tone audiometry revealed pure conductive loss with an air conduction loss of 30 dB. A computed tomography (CT) scan revealed a well-circumscribed strip of solid mass in the right external auditory canal without bony erosion and a medium-density area in the tympanic antrum (Fig. 2). When the mass was excised via endoscopic surgery, some pink fluid was exuded, although an integrated tympanic membrane and normal canal wall were observed. Histopathologic findings showed nest, cord, gland and trabecular proliferations of orbicular-ovate cells under the squamous epithelial layers; some irregular cells located around the nest were found to have vesicular nuclei, eosinophilic cytoplasm and mitotic morphologies (Fig. 3). Immunohistochemistry revealed strongly positive staining for cytokeratin (CK) and neuron-specific enolase (NSE) (Fig. 4) and moderately positive staining for vimentin, showing a pattern consistent with neuroendocrine tumors.

## Discussion

The cNEC is also called Merkel carcinoma because the tumor cell is derived from epidermal Merkel cells. The primary focus of cNEC on the external ear is very rare. To the best of our knowledge, only 24 cases of external ear cNEC have been reported, and only 4 cases were found to originate from the external auditory canal.^4^, ^5^, ^6^

In our patient, the symptoms included itching, intermittent pain, a watery secretion from the right external auditory canal, and hearing loss due to the neuroendocrine nature of cNEC, whereas the cases of cNEC reported by Wang^7^ and Li^6^ were described as a painless neoplasm in the external auditory canal with hearing loss. An orbicular-ovate, pink, firm neoplasm with a smooth surface is the characteristic morphology of cNEC reported in multiple studies, which is in agreement with our case. The clinical tests (particularly CT results) reported by other groups have shown conductive hearing loss and a regularly shaped high-density area in the external auditory canal, which is in agreement with our case study.

Diagnosis is usually delayed because the clinical characteristics of this disease mimic more common otologic conditions, such as adenoid cystic carcinoma.^8^ Malignant tumors should be considered if anti-inflammatory therapies provide no relief. Biopsies should be performed especially when the neoplasm has an orbicular-ovate, pink, firm appearance with a smooth surface that bleeds after minimal contact. The diagnosis rate may be improved if the aforementioned clinical characteristics receive more attention.

The diagnosis and staging of cNEC primarily rely on pathological examination. Tissue analysis may reveal a trabecular distribution of moderately differentiated cells, showing clustered proliferation in the propial or dermal layer, pleomorphic nuclei and inconspicuous nucleoli. Furthermore, frequent mitoses can be seen in hematoxylin and eosin-stained sections. Pyknotic nuclei, suggestive of apoptosis, may also be found in the scattered cell fragments. In our case, immunohistochemistry revealed that the tumor cells expressed cytokeratin, NSE, and vimentin.

A wide local excision with a clear margin is recommended for the treatment of cNEC, with adjunct chemotherapy and radiation therapy. For those patients who cannot tolerate wide surgical excision, radical radiotherapy may be used solely as an effective curative approach against cNEC according to Harrington.^9^ Adjunct radiotherapy for primary tumors and regional lymph nodes has been shown to result in better local regional control and disease-free survival.^10^ Chemotherapeutics are used in cases of locally advanced disease or for tumors that cannot be completely resected, as a palliative measure or in cases of recurrence. However, the use of adjuvant chemotherapy is associated with decreased survival compared to not receiving chemotherapy.^11^ The primary tumor in our case study was classified at a low disease stage, such that local excision was applied without the consideration of chemotherapy. However, the diagnosis of cNEC was uncertain until a post-surgical biopsy was performed. Adjunct radiotherapy was used due to the concern that the resection margins were not safe enough. In this case, the follow-up lasted for 12 months, with no recrudescence or metastasis observed. From our perspective, a comprehensive therapy that includes local excision with functional structure protection and radiotherapy is a novel and effective approach to treating cNEC, which may avoid the dysfunction of important anatomic structures after wide excision.

## Conclusion

Cutaneous neuroendocrine carcinoma remains an aggressive tumor with a poor prognosis. Preoperative diagnosis and accurate staging are important in ensuring adequate treatment. Because of the rarity of this disease, a consensus on the diagnosis and treatment of cNEC in the external auditory canal has not been reached. We illustrate that cNEC should be considered in the differential diagnosis when a round and firm tumor with a smooth surface is seen in the external auditory canal with no or minor symptoms. Pathology is the most confirmative diagnosis. Tumor resection with adjuvant radiotherapy is recommended for the consideration and protection of important structures.

## Conflicts of interest

The authors declare no conflicts of interest.
